# The role of antiangiogenic monoclonal antibodies combined to EGFR-TKIs in the treatment of advanced non-small cell lung cancer with activating EGFR mutations: acquired resistance mechanisms and strategies to overcome them

**DOI:** 10.20517/cdr.2022.77

**Published:** 2022-11-02

**Authors:** Danilo Rocco, Luigi Della Gravara, Giovanni Palazzolo, Cesare Gridelli

**Affiliations:** ^1^Department of Pulmonary Oncology, AORN dei Colli Monaldi, Naples 80131, Italy.; ^2^Department of Experimental Medicine, Università degli studi della Campania “Luigi Vanvitelli”, Naples 80138, Italy.; ^3^UO Oncologia, “ULSS 6 Euganea”, Cittadella (PD) 35131, Italy.; ^4^Division of Medical Oncology, “S.G. Moscati” Hospital, Avellino 83100, Italy.

**Keywords:** NSCLC, EGFR, T790M, MET, bevacizumab, ramucirumab, TKI, resistance mechanisms

## Abstract

As of today, only two antiangiogenic monoclonal antibodies plus epidermal growth factor receptor-tyrosine kinase inhibitor (EGFR-TKI) combinations are FDA and EMA-approved and are recommended by American Society of Clinical Oncology, European Society for Medical Oncology, and National Comprehensive Cancer Network for the first-line treatment of EGFR+ advanced non-small cell lung cancer patients: erlotinib plus bevacizumab and erlotinib plus ramucirumab. However, all treated patients eventually become unresponsive to such drugs, due to several different acquired resistance mechanisms, mainly represented by T790M substitutions and MET amplifications. While osimertinib treatment in T790M+ patients still represents the only approved treatment, MET-TKIs will likely change this status quo in the near future. In fact, existing clinical data strongly support a role for MET-TKI-based combinations in EGFR+ MET-amplified patients, possibly revolutionizing our current treatment algorithm. Chemotherapy plus immunotherapy plus antiangiogenic therapy combinations could also represent another useful addition.

## INTRODUCTION

### Epidemiology of EGFR+ NSCLC and preclinical background for antiangiogenic monoclonal antibody plus EGFR-TKI combinations

According to the most recent GLOBOCAN data, in 2020, lung cancer accounted for approximately 2,200,000 new cases and 1,800,000 deaths^[1]^. Overall, 85% of these cases are represented by non-small cell lung cancer (NSCLC), and the majority of NSCLC are histologically represented by adenocarcinomas (55%)^[2]^. With respect to genetic alterations, the vast majority of currently druggable mutations are diagnosed in adenocarcinomas, and specifically EGFR (epidermal growth factor receptor)-activating mutations (exon 19 deletion and exon 21 L858R substitution) are among the most common ones, accounting for approximately 15% of non-squamous NSCLC cases in North American and European patients and 40%-50% of non-squamous NSCLC in Asian patients^[3]^.

With reference to EGFR mutations, vast literature data emphasize the link between EGFR axis hyperactivity and VEGF (vascular endothelial growth factor) induction and upregulation, as well as VEGFR (vascular endothelial growth factor receptor)-EGFR synergy, in promoting tumor growth, thus providing a strong preclinical background for the potential benefits of antiangiogenic monoclonal antibody plus EGFR-tyrosine kinase inhibitor combinations in this subset of patients^[4-6]^.

## CURRENT AND FUTURE CLINICAL PERSPECTIVES

### Regulatory approvals and current guidelines involving antiangiogenic monoclonal antibody plus EGFR-TKI combinations

As of today, only two antiangiogenic monoclonal antibodies plus EGFR-TKI (tyrosine kinase inhibitor) combinations are FDA (US Food and Drug Administration) and EMA (European Medicines Agency) approved and ASCO (American Society of Clinical Oncology), ESMO (European Society for Medical Oncology), and NCCN (National Comprehensive Cancer Network) recommended for the first-line treatment of EGFR+ advanced NSCLC patients: erlotinib (EGFR TKI) plus bevacizumab (anti-VEGF-A mAb) and erlotinib plus ramucirumab (anti-VEGFR2 mAb)^[7-10]^.

### Pivotal trials involving antiangiogenic monoclonal antibody plus EGFR-TKI combinations

The erlotinib plus bevacizumab combination received regulatory approval and clinical recommendation thanks to the results coming from several different phase II and III trials. In the single-arm phase II BELIEF trial, 109 European naïve non-squamous advanced NSCLC patients with EGFR activating mutations were administered an erlotinib plus bevacizumab combination, achieving an overall PFS (progression-free survival) of 13.2 months and a manageable safety and tolerability profile with grade 4 TRAEs (treatment-related adverse events) in 4% of treated patients^[11]^.

In the randomized controlled phase II JO25567 study, 154 Japanese naïve non-squamous advanced NSCLC patients with EGFR activating mutations were randomized 1:1 to receive erlotinib plus bevacizumab or erlotinib as monotherapy. The combination arm showed superior results with mPFS of 16.0 months *vs*. 9.7 months and HR (hazard ratio) for progression or death of 0.54 and comparable safety and tolerability profile with serious TRAEs in 24% of treated patients *vs*. 25% of treated patients^[12]^. However, after an extended follow-up of 34.7 months, while the combination arm continued to show superior results in terms of PFS at 16.4 months *vs*. 9.8 months (HR for progression or death, 0.52), no significant benefit in terms of OS (overall survival) was observed at 47.0 months *vs*. 47.4 months (HR for death, 0.81; *P = *0.3267), and no new safety issues were reported^[13]^.

Similarly, in the randomized controlled phase III NEJ026 trial, 228 Japanese naïve non-squamous advanced NSCLC patients with EGFR activating mutations were randomized 1:1 to be given erlotinib plus bevacizumab or erlotinib alone. At a first analysis, after a median follow-up of 12.4 months, the PFS results favored the combination arm at 16.9 months *vs*. 13.3 months (HR for progression or death, 0.60), and a manageable safety profile was observed with serious TRAES in 8% of treated patients *vs*. 4% of treated patients^[14]^. However, once again, at a longer follow-up of 39.2 months, no significant benefit in terms of OS was reported between the combination and monotherapy arms at 50.7 months *vs*. 46.2 months (HR for death, 1.007; *P = *0.97)^[15]^.

More recently, these conclusions have also been supported by the BEVERLY study. In the phase III randomized controlled BEVERLY trial, 160 Italian naïve advanced NSCLC patients with EGFR activating mutations were randomized 1:1 to receive erlotinib plus bevacizumab or erlotinib as monotherapy. After 31 months of median follow-up, a clear advantage was reported with respect to PFS at 15.4 months *vs*. 9.7 months (HR for death or progression, 0.60) but not with respect to OS at 28.4 months *vs*. 23.0 months (HR for death, 0.70; *P = *0.12)^[16,17]^.

Even more recently, the results from the randomized controlled phase III ARTEMIS-CTONG1509 trial also seem to be in line with the above-mentioned data. In this trial, 311 Chinese naïve advanced NSCLC patients with EGFR activating mutations were randomized 1:1 to be administered erlotinib plus bevacizumab or erlotinib alone. Once more, favorable PFS data were reported for the experimental arm at 17.9 months *vs*. 11.2 months (HR for death or progression, 0.55), but OS did not manage to reach significant superiority at 36.2 months *vs*. 31.6 months (HR for death, 0.92; *P = *0.581)^[18]^.

Ramucirumab received regulatory approval and clinical recommendation thanks to the data from the RELAY study. In this randomized controlled phase III trial, 449 European and Asian naïve advanced NSCLC patients with EGFR activating mutations were randomized 1:1 to receive erlotinib plus ramucirumab or erlotinib as monotherapy. At the pre-specified data cut-off after a median follow-up of 20.7 months, the combination arm performed better than the control one in terms of PFS at 19.4 months *vs*. 12.4 months (HR for death or progression, 0.59), albeit with some safety concerns with grade 3-4 TRAEs in 72% of treated patients *vs*. 54% of treated patients. OS data are still awaited^[19-21]^. In addition, following subgroup analysis, extremely interesting results were reported about TP53- and L858R-mutated patients. While EGFR+ TP53+ patients are classically associated with a worse outcome^[22]^, they benefited from the experimental combination in terms of mPFS at 15.2 months *vs*. 10.6 months (HR for death or progression, 0.54); in particular, the HR data prove to be superior to those of ITT (0.59)^[23]^. Similarly, the L858R patients presented particularly favorable mPFS results at 19.4 months *vs*. 11.2 months (HR for death or progression, 0.618)^[24]^. It is worth mentioning that this result is superior to the one reported in the FLAURA trial (osimertinib *vs*. gefitinib or erlotinib in naïve EGFR+ advanced NSCLC patients) at 18.9 months *vs*. 10.2 months^[25]^. These results are particularly relevant because, while osimertinib (a third-generation EGFR-TKI) represents the most used and effective treatment in naïve EGFR+ advanced NSCLC patients, erlotinib plus ramucirumab combination could represent a more useful and attractive choice in these two selected subsets of patients. To further investigate this matter, a phase III trial comparing erlotinib plus ramucirumab to osimertinib monotherapy in naïve EGFR+ L858R advanced NSCLC patients is currently ongoing^[26]^.

### Acquired resistance mechanisms to antiangiogenic monoclonal antibody plus EGFR-TKI combinations

As the above-mentioned data extensively show, antiangiogenic monoclonal antibody plus EGFR-TKI combinations have managed to grant excellent results in terms of survival and safety, comparable with those of EGFR-TKI monotherapy [Table 1]. This is especially true when we compare these results with those granted by standard chemotherapy and/or immunotherapy regimens^[27-29]^. However, even with these treatments, the progression of disease is inevitable. In fact, all the treated patients eventually become unresponsive to such drugs due to several different acquired resistance mechanisms that have been best investigated in patients treated with erlotinib with or without bevacizumab. Thanks to the vast literature data, it is now well established that the main acquired resistance mechanism in erlotinib-treated patients is represented by exon 20 T790M substitution (55%-60% of cases), followed by MET amplification (5), HER2 amplification (5%-10%), SCLC (small cell lung cancer) transformation (2%-10%), and epithelial-mesenchymal transition (2%-10%)^[30-33]^.

**Table 1:** Overview of data from trials exploring approved antiangiogenic mAbs plus EGFR-TKI combinations for the treatment of aNSCLC with activating EGFR mutations

**Name of the trial** **(number of patients)**	**Phase of the trial**	**Experimental arm**	**Control arm**	**Efficacy data in months**
BELIEF (*n = *109)	II	Erlotinib + bevacizumab	/	mPFS: 13.2
JO25567 (*n = *154)	II	Erlotinib + bevacizumab	Erlotinib	mPFS: 16.4 *vs*. 9.8 (HR: 0.54) mOS: 47.0 *vs*. 47.4 (HR: 0.81; *P = *0.3267)
NEJ026 (*n = *228)	III	Erlotinib + bevacizumab	Erlotinib	mPFS: 16.9 *vs*. 13.3 (HR: 0.60) mOS: 50.7 *vs*. 46.2 (HR: 1.007; *P = *0.97)
BEVERLY (*n = *160)	III	Erlotinib + bevacizumab	Erlotinib	mPFS: 15.4 *vs*. 9.7 (HR: 0.60) mOS: 28.4 *vs*. 23.0 (HR: 0.70; *P = *0.12)
ARTEMIS-CTONG1509 (*n = *311)	III	Erlotinib + bevacizumab	Erlotinib	mPFS: 17.9 *vs*. 11.2 (HR: 0.55) mOS: 36.2 *vs*. 31.6 (HR: 0.92; *P = *0.581)
RELAY (*n = *449)	III	Erlotinib + ramucirumab	Erlotinib	mPFS: 19.4 *vs*. 12.4 (HR: 0.59)

EGFR-TKI: Epidermal growth factor receptor-tyrosine kinase inhibitor; NSCLC: non-small cell lung cancer; HR: hazard ratio.

The same applies to erlotinib plus bevacizumab-treated patients, albeit with different incidences. In fact, these patients seem to present lower rates of these mutations: T790M substitutions (35%-45% of cases), MET amplifications (3%-6%), HER2 amplification (2%-5%), and SCLC transformation (3%-5%)^[18,34,35]^.

### Current strategies and future perspectives in overcoming acquired resistance mechanisms

As of now, the only FDA- and EMA-approved and ASCO-, ESMO-, and NCCN-recommended treatment for EGFR+ advanced NSCLC patients progressing after EGFR-TKI therapy is osimertinib, the administration of which is limited to T790M+ patients. No approved agents are available for EGFR+ MET-amplified advanced NSCLC patients progressing after EGFR-TKI therapy, and platinum-based chemotherapy still represents the standard of care in this subset of patients^[7-10]^.

Osimertinib received regulatory approval and clinical recommendation following the results from the AURA 3 trial. In this randomized controlled phase III trial, 419 EGFR+ T790M+ advanced NSCLC patients whose disease had progressed after first-line EGFR-TKI treatment were randomized 2:1 to receive osimertinib or cis/carboplatin plus pemetrexed followed by pemetrexed maintenance. At data cut-off, the osimertinib arm clearly outperformed the control one in terms of both response and survival with ORR (objective response rate) of 71% of treated patients *vs*. 31% of treated patients, PFS of 10.1 months *vs*. 4.4 months (HR for death or progression, 0.30) as well as in terms of safety and tolerability with grade ≥ 3 TRAEs in 23% of treated patients *vs*. 47% of treated patients^[36]^. However, in the final analysis, osimertinib did not show a significant benefit in terms of OS at 26.8 months *vs*. 22.5 months (HR for death, 0.87; *P = *0.277). However, this result seems to be linked to the very high rate of crossover from platinum plus pemetrexed to osimertinib (73% of platinum-treated patients). In fact, very different results were reported after adjusting OS data for crossover: 26.8 months *vs*. 15.9 months (HR for death, 0.54)^[37]^. On a side note, it is worth mentioning that the main acquired resistance mechanism in patients progressing on second-line osimertinib is represented by MET amplification, accounting for 5%-50% of all cases^[38]^. The addition of bevacizumab to osimertinib failed to show meaningful PFS or OS improvement in both T790M+ pre-treated patients and naïve patients^[39,40]^.

With respect to EGFR+ MET-amplified advanced NSCLC patients progressing after EGFR-TKI therapy, even though no drugs are approved yet, several encouraging trials have already assessed and are currently further investigating the safety and efficacy of different MET-TKIs, savolitinib, tepotinib, and capmatinib being the most promising ones^[41]^. In the phase Ib NCT02374645 trial, the savolitinib plus gefitinib (EGFR TKI) combination was administered to 57 Chinese EGFR+ MET-amplified advanced NSCLC patients progressing after EGFR-TKI therapy, reporting a manageable safety profile with grade ≥ 3 TRAEs in 27% of treated patients and reasonable ORR in T790M patients (52% of treated patients) and patients whose T790M state was unknown (40% of treated patients)^[42]^.

Another savolitinib-based combination (osimertinib plus savolitinib) was investigated in the phase Ib TATTON trial. All 180 included patients were EGFR+ MET-amplified advanced NSCLC patients progressing after EGFR-TKI therapy. Patients in Cohort B1 were pre-treated with osimertinib; patients in Cohort B2 were not pre-treated with osimertinib and were T790M-; patients in Cohort B3 were not pre-treated with osimertinib and were T790M+; and patients in Cohort D were not pre-treated with osimertinib, but T790M-, and received a smaller dose of savolitinib. No dose-limiting toxicities were reported, 57% of patients in Cohort B and 38% of patients in Cohort D experienced grade 3 or worse TRAEs, and partial responses were reported in 48% of patients in Cohort B and 64% of patients in Cohort D^[43]^.

The randomized controlled phase II NCT04606771 trial is currently recruiting EGFR+ MET-amplified advanced NSCLC patients progressing after osimertinib therapy to be randomized 1:1 to receive savolitinib plus osimertinib or savolitinib plus placebo. ORR is the primary objective, and the study completion date is estimated to be February 29, 2024^[44]^.

Tepotinib was first investigated in combination with gefitinib in 55 Asian EGFR+ advanced NSCLC patients with acquired resistance to a previous EGFR-TKI (MET-amplification or overexpression) in the randomized phase Ib/II INSIGHT study. Even though this trial failed to show favorable survival results in the intention to treat population and was thus terminated early, extremely promising results were reported for the MET-amplified population: PFS of 16.6 months *vs*. 4.2 months (HR for death or progression, 0.13) and OS of 37.3 months *vs*. 13.1 months (HR for death, 0.08)^[45]^. These results were further confirmed in the final analysis: PFS of 19.3 months *vs*. 5.5 months (HR for death or progression, 0.18) and OS of 37.3 months *vs*. 13.1 months (HR for death, 0.08)^[46]^. Tepotinib with or without osimertinib is currently being investigated in the phase II INSIGHT 2 trial in EGFR+ MET-amplified advanced NSCLC patients progressing after osimertinib therapy. Recruitment is currently ongoing, dose-limiting toxicities and ORR are the primary objectives, and the study completion date is estimated to be March 30, 2023^[47]^.

Finally, capmatinib was assessed in combination with gefitinib in the phase Ib/II NCT01610336 study, in which 161 EGFR+ advanced NSCLC patients with acquired resistance to a previous EGFR-TKI (MET-amplification or overexpression) received a capmatinib plus gefitinib combination therapy. Modest results were reported for the intention to treat the population with an ORR of 27%; however, remarkable results were reported for MET-amplified patients with an ORR of 47%^[48,49]^. The randomized controlled phase III GEOMETRY-E trial is currently recruiting EGFR+ MET-amplified T790M- advanced NSCLC patients progressing after EGFR-TKI treatment to be randomized 1:1 to receive a capmatinib plus osimertinib combination or cis/carboplatin plus pemetrexed followed by pemetrexed maintenance. Dose-limiting toxicities and PFS are the primary objectives and the study completion date is estimated to be March 30, 2027^[50]^.

On a more general side note, it is worth mentioning that, in the recent IMpower150 trial, building on promising preclinical data^[51]^, the carboplatin plus paclitaxel plus bevacizumab plus atezolizumab combination managed to be the first and currently only association of chemotherapy plus immunotherapy plus antiangiogenic therapy to show favorable results in EGFR-TKI-pretreated EGFR+ aNSCLC patients. In fact, when compared to the control arm (carboplatin plus paclitaxel plus bevacizumab), the former combination managed to outperform the latter with mOS of 27.8 months *vs*. 18.1 months (HR for death, 0.74)^[52]^, thus representing another promising strategy in overcoming resistance mechanisms.

## CONCLUSION

With reference to the main known acquired resistance mechanisms to the antiangiogenic monoclonal antibody plus EGFR-TKI combinations in EGFR+ advanced NSCLC patients (T790M substitution and MET amplification), we can report that, while osimertinib treatment in T790M+ patients still represents the only approved treatment, MET-TKIs will likely change this status quo in the near future. In fact, the extensive above-mentioned clinical data strongly support a role for MET-TKI-based combinations in EGFR+ MET-amplified patients, possibly revolutionizing our current treatment algorithm [Table 2]. Chemotherapy plus immunotherapy plus antiangiogenic therapy combinations could also represent another useful addition.

**Table 2:** Ongoing trials investigating MET-TKI-based combinations in advanced NSCLC patients

**Clinical trial name**	**Phase**	**Subset of patients**	**Experimental arm**	**Control arm**	**Primary objective(s)**	**Study completion date**
NCT04606771	II	EGFR+ MET-amplified progressing after osimertinib	Savolitinib + osimertinib	Savolitinib + placebo	ORR	February 29, 2024
INSIGHT 2	II	EGFR+ MET-amplified progressing after osimertinib	Tepotinib ± osimertinib	No control arm	Dose-limiting toxicities and ORR	March 30, 2023
GEOMETRY-E	III	EGFR+ MET-amplified T790M- progressing after EGFR TKI	Capmatinib + osimertinib	Cis/carboplatin + pemetrexed followed by a pemetrexed maintenance	Dose-limiting toxicities and PFS	March 30, 2027

MET-TKI: Mesenchymal epithelial transition-tyrosine kinase inhibitor; EGFR: epidermal growth factor receptor; NSCLC: non-small cell lung cancer; ORR: objective response rate.

## DECLARATIONS

### Copyright

© The Author(s) 2022.
